# End-to-end deep learning approach to mouse behavior classification from cortex-wide calcium imaging

**DOI:** 10.1371/journal.pcbi.1011074

**Published:** 2024-03-13

**Authors:** Takehiro Ajioka, Nobuhiro Nakai, Okito Yamashita, Toru Takumi

**Affiliations:** 1 Department of Physiology and Cell Biology, Kobe University School of Medicine, Chuo, Kobe, Japan; 2 Department of Computational Brain Imaging, ATR Neural Information Analysis Laboratories, Seika, Kyoto, Japan; 3 RIKEN Center for Biosystems Dynamics Research, Chuo, Kobe, Japan; Northwestern University Feinberg School of Medicine, UNITED STATES

## Abstract

Deep learning is a powerful tool for neural decoding, broadly applied to systems neuroscience and clinical studies. Interpretable and transparent models that can explain neural decoding for intended behaviors are crucial to identifying essential features of deep learning decoders in brain activity. In this study, we examine the performance of deep learning to classify mouse behavioral states from mesoscopic cortex-wide calcium imaging data. Our convolutional neural network (CNN)-based end-to-end decoder combined with recurrent neural network (RNN) classifies the behavioral states with high accuracy and robustness to individual differences on temporal scales of sub-seconds. Using the CNN-RNN decoder, we identify that the forelimb and hindlimb areas in the somatosensory cortex significantly contribute to behavioral classification. Our findings imply that the end-to-end approach has the potential to be an interpretable deep learning method with unbiased visualization of critical brain regions.

## Author summary

Deep learning is used in neuroscience, and it has become possible to classify and predict behavior from massive data of neural signals from animals, including humans. However, little is known about how deep learning discriminates the features of neural signals. In this study, we perform behavioral classification from calcium imaging data of the mouse cortex and investigate brain regions important for the classification. By the end-to-end approach, an unbiased method without selecting regions of interest, we clarify that information on the somatosensory areas in the cortex is important for distinguishing between resting and moving states in mice. This study will contribute to the development of interpretable deep-learning technology.

## Introduction

Neural decoding is a method to understand how neural activity relates to perception systems and the intended behaviors of animals. Deep learning is a powerful tool for accurately decoding movement, speech, and vision from neural signals from the brain and for neuroengineering such as brain-computer interface (BCI) technology that utilizes the correspondence relationship between neural signals and their intentional behavioral expressions [1–3]. In clinical studies, electrical potentials measured by implanted electrodes in a specific brain area, such as the motor cortex, were often used to decode the intended movements such as finger motion, hand gesture, and limb-reaching behavior [4–7]. In contrast, neural decoding for whole-body movements such as running and walking remains uncertain due to technical difficulties. For example, contamination of noise signals (e.g., muscular electrical signals during muscular contraction) detected in electroencephalography (EEG) recording disturbs the decoding of behaviors, and the immobilized conditions in functional magnetic resonance imaging (fMRI) and magnetoencephalography (MEG) scanners prevent neural recording for whole-body movement. It is challenging to decode voluntary behaviors during whole-body movements from brain dynamics that contain complex information processing from motor planning to sensory feedback.

The calcium imaging technique allows us to measure *in vivo* neural activity during behavioral conditions from microscopic cellular to mesoscopic cortex-wide scales [8,9]. Recent studies suggest that cellular activities have enough resolution for decoding behaviors. The cellular imaging data using microendoscopy in the hippocampal formation was used to decode free-moving mouse behaviors [10–12] by a Baysian- and a recurrent neural network (RNN)-based decoders. In addition, a convolutional neural network (CNN) is also used to predict the outcome of lever movements from microscopic images of the motor cortex in mice [13]. On the other hand, it is little known whether mesoscopic cortex-wide calcium imaging that contains neural activity at the regional population- but not the cellular resolution is applicable for neural decoding of animal behaviors. Our recent study suggests the potential to classify mouse behavioral states from mesoscopic cortex-wide calcium imaging data using a support vector machine (SVM) classifier [14]. This mesoscopic strategy may be appropriate for end-to-end analyses since it deals with substantial spatiotemporal information of neural activity over the cortex.

Preprocessing calcium imaging data, encompassing actions such as downsampling spatiotemporal dimensions and selecting specific regions of interest (ROIs) within the images, can refine data and generally enhance decoder performance, whereas it may also obscure valuable spatiotemporal information. Conversely, employing images with minimal to no processing preserves the integrity of the original data, facilitating more immediate decoding capabilities. This approach is suitable for near real-time behavior decoding and identification of significant image areas for neural decoding without arbitrary data handling. CNN most applies to image data, while RNN is often used for sequential inputs, including time-variable data [2]. By combining these architectures, CNN-RNN decoders better capture temporal dynamics of behavioral features such as hand and finger movements from intracortical microelectrode array, electrocorticography, and electromyogram recordings, compared with classical machine learning methods [6,7,15,16]. Given these technological advances, we designed a two-step CNN-RNN model for decoding mouse behavioral states from the mesoscopic cortical fluorescent images without intermediate processing. Moreover, it is desired to identify biologically essential features for deep learning classification to make the models interpretable and transparent for explanations of neural decoding as suggested by XAI-Explainable Artificial Intelligence [17]. To this end, we applied a visualization strategy to identify the features that contributed to the performance of the CNN-RNN-based classifications for our calcium imaging data [18], which was applied to electrophysiology in the neuroscience field [19]. We identified the somatosensory areas are the most significant features for the type of behavioral states during voluntary locomotion behavior. This unbiased identification was supported by separate analyses of regional cortical activity using deep learning with RNN and the assessment by Deep SHAP, a developed Shapley additive explanations (SHAP) for deep learning [20,21]. Our findings demonstrate possibilities for neural decoding of voluntary behaviors with the whole-body motion from the cortex-wide images and advantages for identifying essential features of the decoders.

## Results

**Fig 1:**
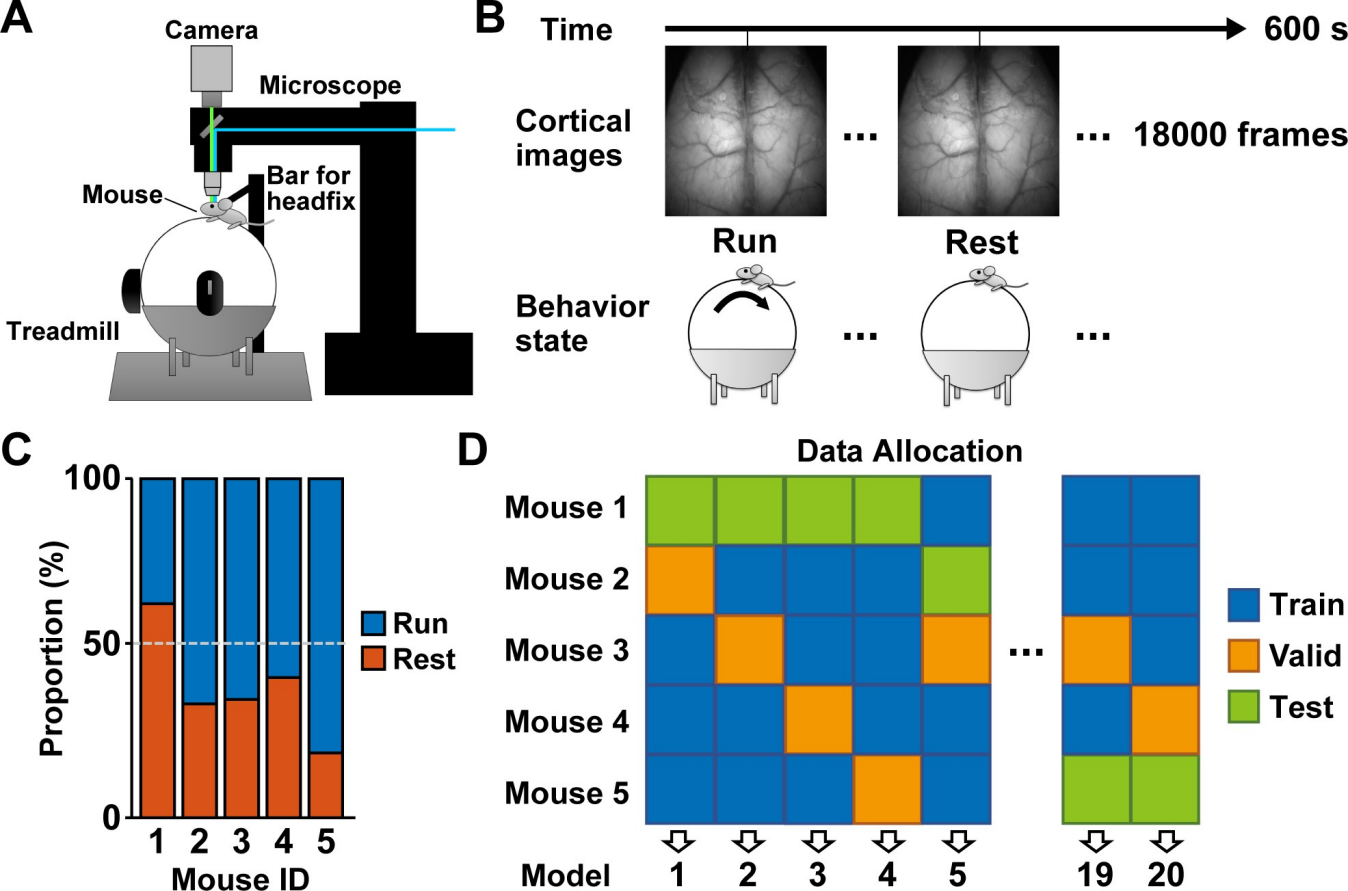
Cortical activity and behavioral states in behaving mice. (A) A schematic illustration of the experimental setup for measuring mesoscopic cortical calcium imaging and locomotor activity. (B) Images were obtained at 30 frames per second during a 600 s session. The label of behavioral state was based on locomotion speed (>0.5 cm/s) at the corresponding frame. (C) Proportions of the behavioral states in each mouse (n = 11–14 sessions from 5 mice). (D) The data allocation on a per-mouse basis. The data of each mouse was assigned at the ratio of 3:1:1 for training (Train), validation (Valid), and testing (Test).

To perform behavior classification from the cortical activity with deep learning, we used the previously reported data composed of mesoscopic cortex-wide 1-photon calcium imaging in the mouse, which exhibits voluntary locomotion behavior in a virtual environment under head-fixed conditions [14]. The fluorescent calcium signals from most of the dorsal cortex were imaged at a frame rate of 30 frames/s during a 10-min session (18,000 frames/session) from behaving mice (**Figs 1A–1B**). Two behavioral states (run or rest) were defined by a threshold of the speed of locomotion (>0.5 cm/s) and binarized as 1 for a run and 0 for rest in each frame. The proportion of all run states during a session differed according to individual mice (mean ± SD; mouse ID1, 36 ± 8% (n = 11 sessions); ID2, 66 ± 22% (n = 12 sessions); ID3, 65 ± 16% (n = 14 sessions); ID4, 58 ± 11% (n = 15 sessions); ID5, 80 ± 8% (n = 12 sessions); **Fig 1C**). We used all image data (1,152,000 images from 64 sessions) for deep learning decoding. To generalize decoding across individuals, we assigned the data to training, validation, and testing at the ratio of 3:1:1 on a per-mouse basis (**Fig 1D**). Thus, we generated 20 models for all combinations and classified the test data with each.

### CNN-based end-to-end deep learning accurately classified behavioral states from functional cortical imaging signals

**Fig 2:**
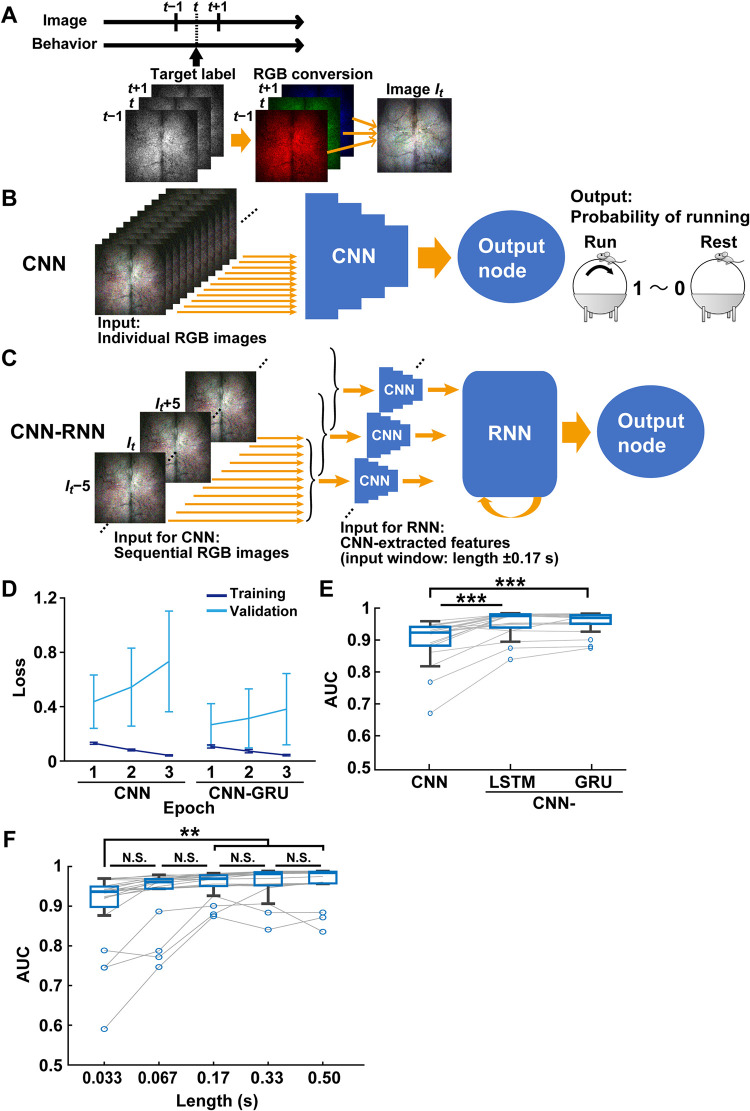
Behavioral state classification using deep learning with CNN. (A) Image preprocessing for deep learning with CNN. An image at frame *t* with images at neighboring frames (frame *t* −1 and *t* +1) was converted to an RGB image (image *I*_*t*_) labeled with the behavioral state. (B) Schematic diagram of the CNN decoder. CNN was trained with individual RGB images. Then, CNN outputs the probability of running computing from the 1,280 extracted features for each image. (C) Schematic diagram of the CNN-RNN decoder. The pre-trained CNN extracted 1,280 features from individual RGB images in the first step. In the second step, a series of 1,280 extracted features obtained from consecutive images (e.g., eleven images from *I*_*t*_ −5 to *I*_*t*_ +5 (= input window, length ±0.17 s)) were input to GRU-based RNN. Then, the RNN output probability of running. (D) Loss of CNN and CNN-GRU during training and validation across three epochs. (E) The area under the receiver operating characteristic curves (AUC) was used to indicate the accuracy of decoders. The performance of decoders with CNN, CNN-LSTM, and CNN-GRU. ***P < 0.001, Wilcoxon rank-sum test with Holm correction, n = 20 models. (F) The performance of CNN-GRU decoders using smaller time windows gradually deteriorated while not above the 0.17 s lengths of the input window. **P < 0.01, N.S., not significant, Wilcoxon rank-sum test with Holm correction, n = 20 models.

We tried to classify the behavioral states from images of cortical fluorescent signals using deep learning with CNN. A pre-trained model for CNN, such as an EfficientNet [22], allows for efficient learning. To handle the single-channel images obtained from calcium imaging, we converted a sequence of three images into a pseudo-3-channel RGB image by combining the previous and next images with the target image (**Fig 2A**). First, we trained CNN with EfficientNet B0, where the individual RGB images were used for input data. The binary behavior labels were used for output (**Fig 2B**). We used the pre-trained model on ImageNet for the initial weight values in training. In training, the loss was reduced by increasing epochs in CNN decoders (**Fig 2D, left**). However, in validation, the loss increased with every epoch (**Fig 2D, left**). These results suggest that when using a large amount of input data (more than 1 million images), CNN learning efficiently progresses even in one epoch, and the models easily fall into overlearning during training. We chose a model with the lowest loss in the validation as a decoder at each data allocation. The decoder’s performance was evaluated by the area under the receiver operating characteristic curve (AUC) for all test data frames. The decoder using CNN alone classified the behavioral states with about 90% accuracy (0.896 ± 0.071, mean ± SD, n = 20 models; **Fig 2E**).

To improve the performance of decoding, we then created a two-step deep learning architecture that combines CNN with long short-term memory- (LSTM) [23] or gated recurrent unit- (GRU) [24] based RNN, in which the output at the final layer of the CNN was compressed by average pooling and connected to the RNN (**Fig 2C**). In this stage, input data was the sequential RGB images from −0.17 s to 0.17 s from the image *t*, located at the center of the input time window. We chose this time window size for decoder tests because the performance has deteriorated when using smaller time windows (see Fig 2F). We used weights of the former CNN decoders for setting the initial values in two-step CNN-RNN. As with CNN decoders, the loss of two-step CNN-RNNs was reduced by the increment of epochs in training, whereas it was increased in validation (**Fig 2D, right**). The performance of behavior state classification was upgraded using two-step CNN-RNNs regardless of individual cortical images and behavioral activities (GRU, 0.955 ± 0.034; LSTM, 0.952 ± 0.041; mean ± SD, n = 20 models; **Fig 2E**). In addition, we confirmed that the classification accuracy slightly deteriorated when using smaller time windows in the two-step deep learning (mean ± SD; 0.033s, 0.896 ± 0.100; 0.067 s, 0.929 ± 0.072; n = 20 models; **Fig 2F**). The performance was gradually improved but not significantly changed when the time windows ranged from 0.17 s to 0.50 s (0.17 s, 0.955 ± 0.034; 0.33 s, 0.960 ± 0.040; 0.50 s, 0.960 ± 0.044; **Fig 2F**). These results demonstrate that deep learning decoding with CNN classifies locomotion and rest states accurately from functional cortical imaging consistently across individual mice, and the performance can be improved by combining it with RNN.

### The somatosensory area contains valuable information on the behavioral classification

**Fig 3:**
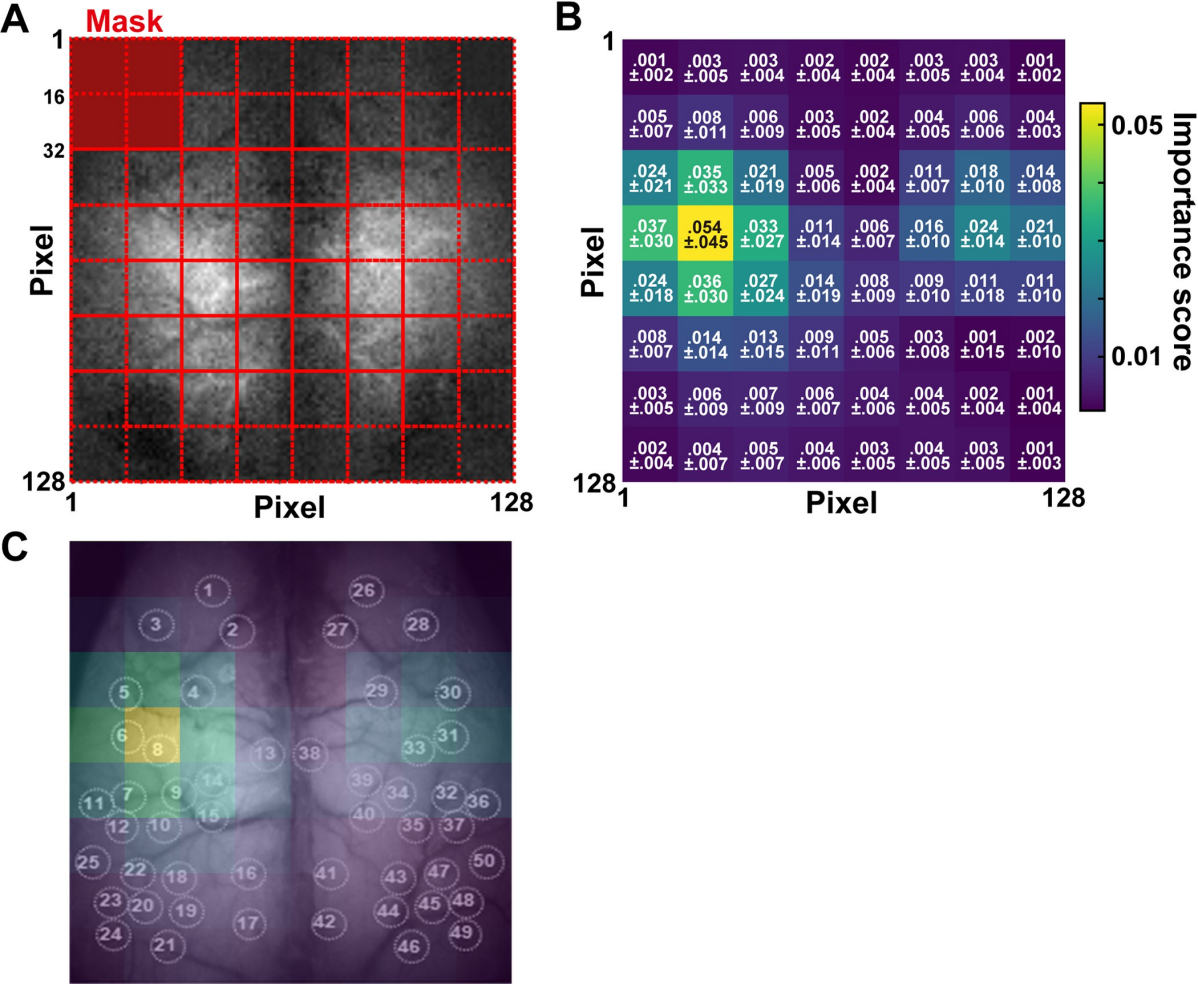
Visualization of essential features in CNN-RNN decoder. (A) An importance score was calculated by averaging differences from classification accuracy using a 1/16 masking area in each image (see Methods for details). (B) Importance scores in each subdivision (mean ± SD, n = 20 models). (C) Overlay of importance scores on the cortical image with ROI positions. See S2 Fig for ROIs 1–50.

To make deep learning decoding interpretable, we tried to quantify the critical areas of images that contributed to the behavioral classification in the CNN-RNN decoder. Zeiler and Fergus proposed the validation method by removing the information of masking areas from images for CNN decoders [18]. Similarly, we calculated and visualized the importance score in subdivisions of images in each decoder using a method named cut-out importance (see Methods for details). Briefly, a subdivision of the image was covered with a mask filled with 0 before evaluation. The decoder tested with the masked images was compared with the decoder tested with the original unmasked images (**Fig 3A**). The importance score indicates how much the decoder’s performance was affected by the masked area. As a result, the highest importance score was detected slightly above the middle of the left hemisphere (0.054 ± 0.045; mean ± SD, n = 20 models; **Fig 3B**). The symmetrical opposite area is also higher than other subdivisions within the right hemisphere (0.024 ± 0.014). This laterality seemed to be derived from individual differences (**S1 Fig**). These subdivisions corresponded to the anterior forelimb and hindlimb areas of the somatosensory cortex (**Fig 3C and S2 Fig**), which were listed in one of the essential cortical areas in our previous study using SVM machine learning classification [14]. When both subdivisions with adjacent areas in the middle left and right hemispheres were occluded simultaneously, the decoding performance was significantly dropped (**S3 Fig**), suggesting that the middle left and right hemispheres are crucial for behavioral classification.

### Regional cortical activity is applicable for the behavioral classification using RNN decoders

To confirm the contribution of the somatosensory cortex in the decoding performance, we designed RNN decoders to classify the behavioral states from activities of the specific cortical areas. For this purpose, the fluorescent signals (*dF/F*), not the inferred spikes of single cells, at 50 regions of interest (ROIs) in the cortex were analyzed as regional cortical activities that accord with known cortical parcellations of the mouse brain (**S2 Fig**) [14]. To reduce baseline fluctuation of cortical activity, we performed data preprocessing by subtracting a 1,000-frame moving average from the normalized fluorescent signals at each ROI (**S4 Fig**).

**Fig 4:**
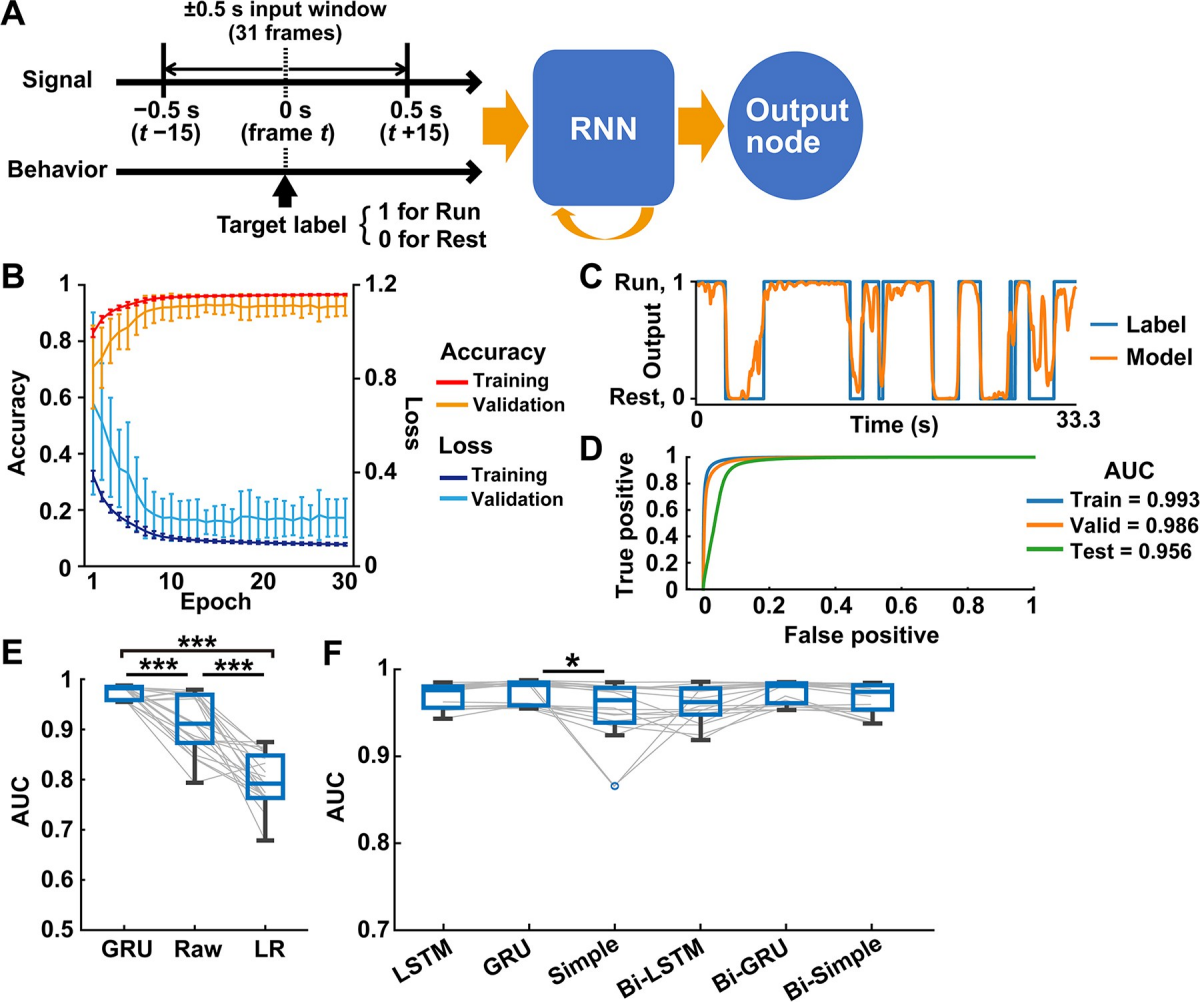
Behavioral state classification from cortical activity using deep learning with RNN. (A) Schematic overview of the RNN decoder for the behavioral state classification. Input is the cortical activities ranging from 0.5 s before (*t*−15 frames) to 0.5 s after (*t*+15 frames) the target frame *t*, which is labeled with a behavior state (1: run, 0: rest). The RNN decoder outputs the probability of behavioral states for all frames of testing data. (B–D) Example of the GRU decoder performance. (B) Learning curve during training and validation across 30 epochs. Loss indicates the cross entropy loss between the outputs and behavioral labels. Accuracy was the percentage of agreement with the label when the output was binarized at a 0.5 threshold. Mean ± SD, n = 20 models. (C) A trace of the output values of a representative decoder and actual behavioral labels in the first 33.3 s of testing data. (D) The receiver operating characteristic curves in the training, validation, and testing data. (E) The performance of GRU decoders trained with preprocessed data (GRU), non-preprocessed data (Raw), and the decoder of the linear regression model (LR). ***P < 0.001, Wilcoxon rank-sum test with Holm correction, n = 20 models. (F) The decoder performance using six types of RNN architectures. LSTM, GRU, simple RNN (Simple), and their bidirectional ones (Bi-). *P < 0.05, Wilcoxon rank-sum test with Holm correction, n = 20 models.

We used a GRU architecture at the beginning of the deep learning decoding with RNN. We set an input window of size 31, including a one-second duration of cortical activity that ranged from −0.5 s (−15 frames) to 0.5 s (+15 frames) from the behavioral state-target label (frame *t*) (**Fig 4A**). To train the deep learning models, we used the ±0.5 s input window with a one-frame sliding window for a total of 1,152,000 frames of data (n = 64 sessions). The random batches of size 256 with Adam optimizer (https://keras.io/api/optimizers/adam/ [25]) and binary cross-entropy loss function were used as model parameters. The models were trained across 30 epochs to converge the loss substantially. In the training data, the loss was reduced in the first 10 epochs, with a slight improvement in the following epochs, and the accuracy was dramatically improved and almost saturated within the first 10 epochs (**Fig 4B**). In the validation, although changes of loss and accuracy behaved similarly, the loss was about twice, and the accuracy was slightly decreased compared to the training (**Fig 4B**). We chose a model with the lowest loss in the validation as a decoder at each data allocation. Then, the decoders classified all frames of the test data into the two behavioral states in good agreement with the behavioral labels (**Fig 4C**), supported by the AUC (**Fig 4D**). The GRU decoder trained with preprocessing data (mean ± SD; GRU, 0.974 ± 0.014; n = 20 each; **Fig 4E**) showed significantly higher performance of behavioral classification than the GRU decoder trained with un-preprocessing data (Raw, 0.911 ± 0.057). Both GRU decoders overcame the performance of a linear regression decoder (LR, 0.797 ± 0.051; **Fig 4E**). The GRU model has the advantage of the temporal dataset, as shown in the previous study comparing machine learning algorithms [26]. The classification performance was a chance level in the control GRU decoder (AUC = 0.492 ± 0.031; mean ± SD; n = 20 models), a null model trained with randomly assigned behavioral labels.

We next examined how much the architectures of RNN affect the decoder performance. All decoders classified behavioral states with high accuracy over 0.95 on average (mean ± SD; LSTM, 0.970 ± 0.013; Simple, 0.953 ± 0.035; Bi-LSTM, 0.960 ± 0.020; Bi-GRU, 0.974 ± 0.012; Bi-Simple, 0.967 ± 0.016; **Fig 4F**), while the simple RNN decoder only underperformed compared with the GRU decoder (*P*<0.05, Wilcoxon rank sum test with Holm correction). Given the accuracy and variance in these decoder performances, GRU and bidirectional GRU architectures are most suitable for the behavioral classification from cortical activity. We used, hereinafter, GRU but not bidirectional GRU as an RNN architecture to simplify the process and time of computing.

**Fig 5:**
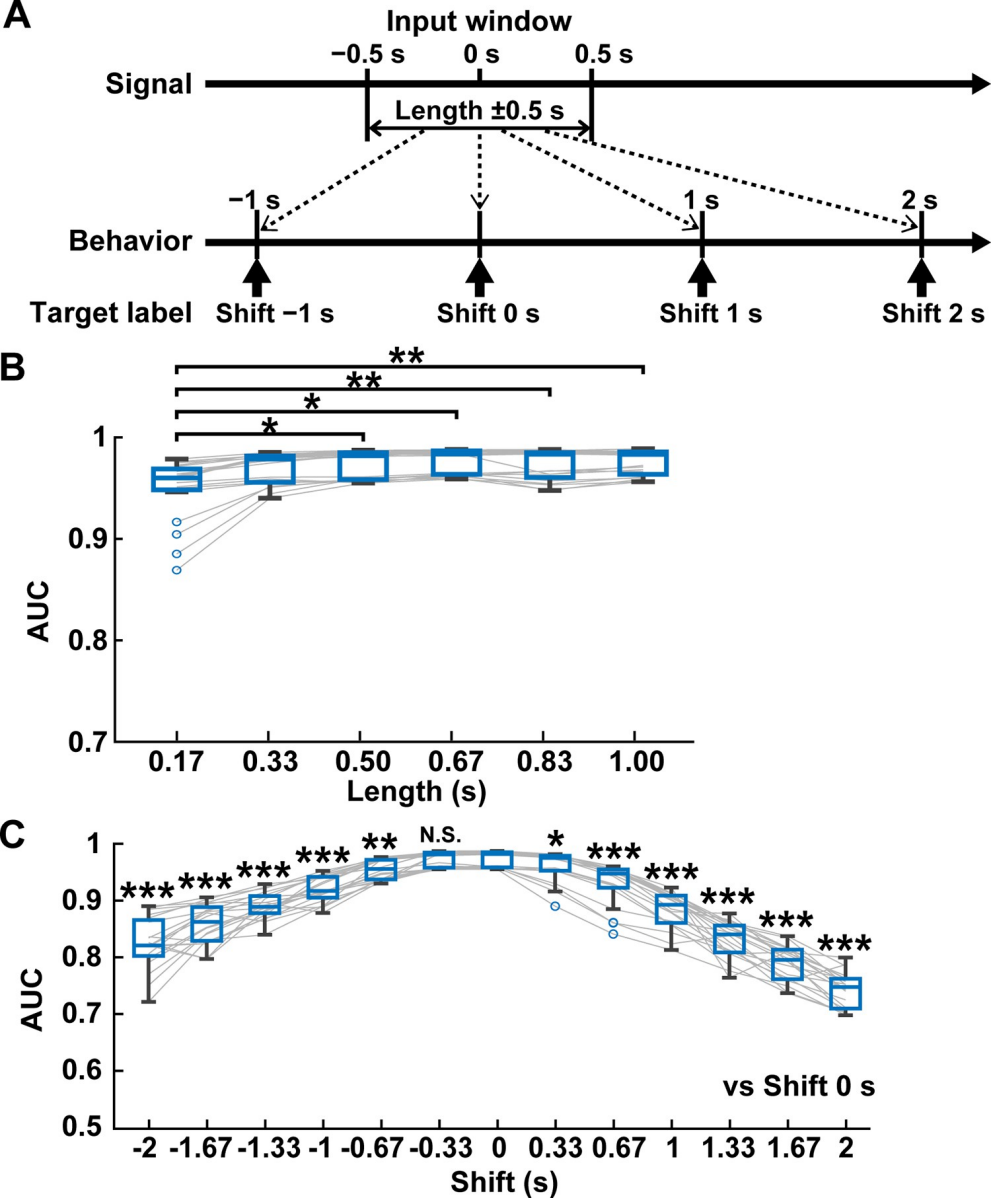
Comparison of input window length and target label’s temporal position. (A) Examples of input window and position of the target labels for behavior classification were shown. “Length” defines the duration of the input window, which ranges arbitral time (e.g., 0.5 s) before and after the center of the input window (0 s). “Shift” defines the temporal location of the target label of behavior classification from the center of the input window. The length 0.5 s and the shift 0 s were used for the criteria for evaluation. (B) The decoder performance of different lengths using a fixed shift 0 s. *P < 0.05, **P < 0.01, Wilcoxon rank-sum test with Holm correction, n = 20 models. (C) The decoder performance of different shifts using a fixed length of 0.5 s. N.S., not significant, *P < 0.05, **P < 0.01, ***P < 0.001, Wilcoxon rank-sum test with Holm correction compared with shift 0 s, n = 20 models.

We investigated whether the temporal specificity of the input data affects the performance of GRU decoders. The initial setting of the length of the input window was 0.5 s when the length contains information on cortical fluorescent signals ranging between 0.5 s before and after the center of the input window (i.e., 0 s). A shift value was set to test which time points of behavioral labels contribute to neural decoding (as shown in Fig 5C). The shift 0 s indicates the position of the behavioral label at 0 s, with no temporal difference between the behavioral label and the input time window (**Fig 5A**). Regarding the analysis of length, the accuracy of the decoder performance from length 0.33 s to 1.0 s did not differ (**Fig 5B**). Only the accuracy was significantly decreased at length 0.17 s, suggesting that a temporally enough length (≥0.33 s) of input window is needed to obtain information on behavioral states from cortical activity. We then examined the temporal distance of the decoding target from the center of the input window by shifting the position of the target labels in a time range from −2 s (backward in time) to 2 s (forward in time) (**Fig 5C**). The accuracy of forward-shifted target labels gradually but significantly decreased with distance from the center of the input window. Similarly, in the back shift of target labels, the performance was significantly degraded when the target labels were set to more than -0.33 s distant from the center of the input window. These results suggest that our decoders are more fitting for predicting current states than future and past states of behaviors.

We also decoded the locomotion speed from regional cortical activity using GRU and linear regression models. The decoding performance of the GRU model (mean ± SD; R^2^ = 0.44 ± 0.12, MAE = 10.4 ± 2.95 cm/s) was superior to the linear regression model (R^2^ = -0.21 ± 0.59, MAE = 17.4 ± 1.12 cm/s; **S5 Fig**) although less than or comparable to the decoders using calcium imaging data from hippocampus at cellular resolution [27,28]. These results suggest that regional cortical activity may include information at fine temporal resolution of behavioral expression.

### Cortical activity in the somatosensory limb areas contributes to the behavioral classification

**Fig 6:**
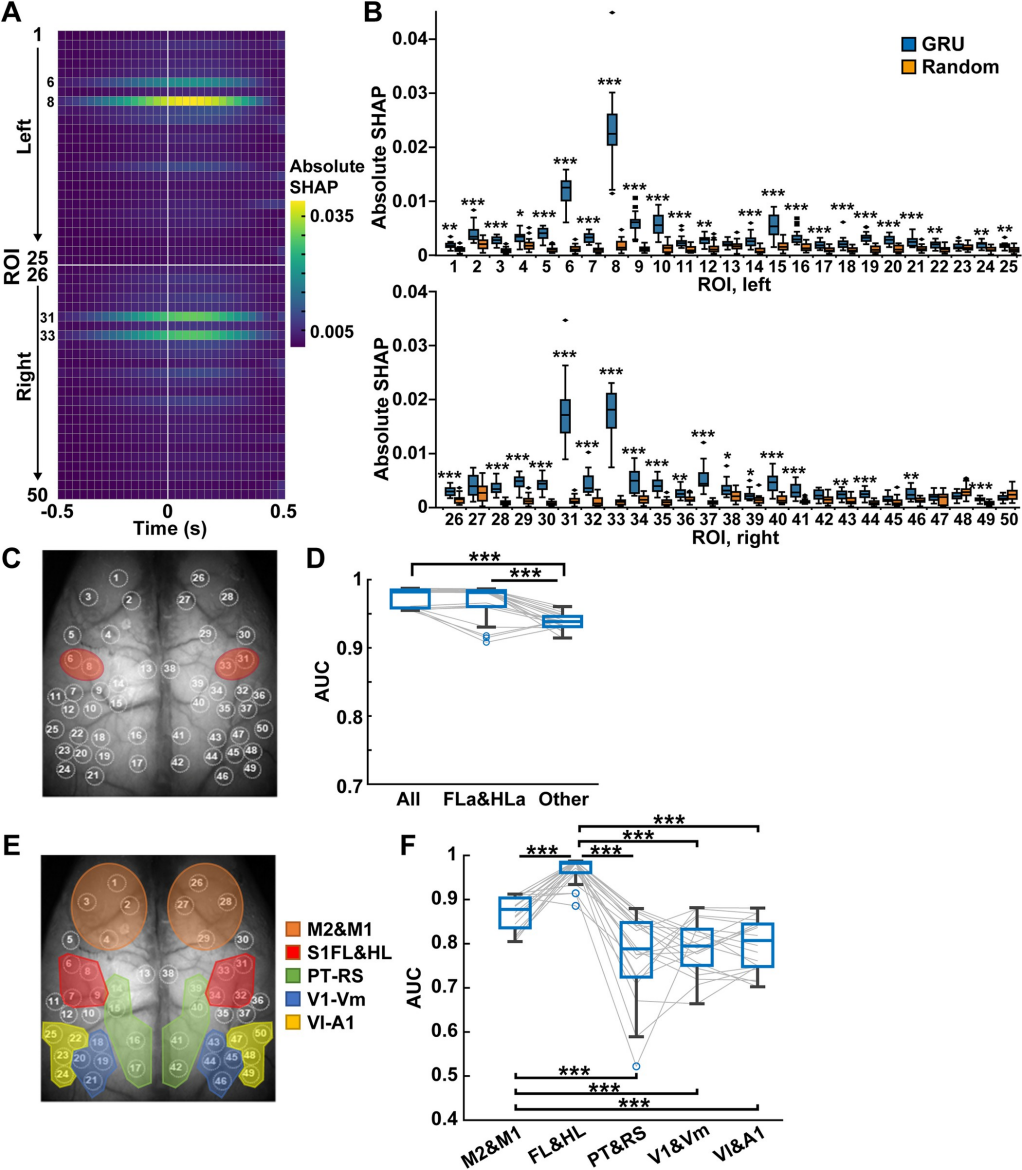
The forelimb and hindlimb areas of the somatosensory cortex contribute to behavioral state classification. (A) The absolute SHAP values at each ROI during the input window across all GRU decoders (50 ROIs × 31 frames (−0.5 ~ 0.5 s) on 20 models average). (B) The absolute SHAP values for all frames at each ROI in GRU decoders with preprocessing data (GRU) and randomly shuffled data (Random). *P < 0.05, **P < 0.01, ***P < 0.001, Wilcoxon rank-sum test with Holm correction, n = 20 models. See S2 Fig for ROIs 1–50. (C) Red ovals indicate the position of the somatosensory cortex anterior forelimb and hindlimb areas (ROIs 6, 8, 31, and 33). (D) Decoder performance using fluorescent signals from all cortical areas (All), somatosensory cortex anterior forelimb and hindlimb areas (FLa&HLa, ROIs 6, 8, 31, and 33), and the other 46 ROIs (Other). ***P < 0.001, Wilcoxon rank-sum test with Holm correction, n = 20 models. (E) The ROIs were divided into five parts: motor areas (M2&M1, ROIs 1–4 and 26–29), somatosensory limb areas (FL&HL, ROIs 6–9 and 31–34), parietal and retrosplenial areas (PT&RS, ROIs 14–17 and 39–42), primary visual and visual medial areas (V1&Vm, ROIs 18–21 and 43–46), and visual lateral and auditory area (Vl&A1, ROIs 22–25 and 47–50). (F) Decoder performance using fluorescent signals from M2&M1, FL&HL, PT&RS, V1&Vm, and Vl&A1. ***P < 0.001, Wilcoxon rank-sum test with Holm correction, n = 20 models.

Finally, we assessed how much cortical areas significantly impact the GRU decoder using Deep SHAP (see Methods for details). We visualized a SHAP value, which is the index to what extent each feature contributes to the behavioral classification in the trained models. The SHAP values in a model were calculated against each input window from ~5% of randomly selected test data. The absolute SHAP values were averaged across all models to quantify the degree of importance in cortical areas (**Fig 6A**). The remarkably high SHAP values were detected in the anterior regions of the somatosensory forelimb (FLa, ROIs 6 and 31) and hindlimb (HLa, ROIs 8 and 33) areas. The peaks of SHAP values were observed around +0.1 s after the center of the input window. Although SHAP values of many cortical areas surpassed those in null models, overall, the magnitudes were smaller than the somatosensory areas (**Fig 6B**).

Based on the results of SHAP, we trained the model using input data only from FLa and HLa (ROIs 6, 8, 31, and 33) and confirmed the performance of the behavioral classification (**Fig 6C**). We masked the signals out of these areas by replacing them with value 0 and used the masked data to train and test the GRU decoder (FLa&HLa). Oppositely, we masked the signals in FLa and HLa with 0 and trained and tested the GRU decoder (Other). The decoder performance using the somatosensory areas was compatible with the decoder trained with all area data (FLa&HLa, 0.966 ± 0.026; mean ± SD, n = 20 models; **Fig 6D**). However, the decoder using other cortical areas underperformed (Other, 0.938 ± 0.011; mean ± SD, n = 20 models; **Fig 6D**).

We further tested the group of cortical areas. We divided bilateral cortical areas into five parts (motor areas (M2&M1, ROIs 1–4, 26–29); somatosensory limb areas (FL&HL, ROIs 6–9, 31–34); parietal and retrosplenial areas (PT&RS, ROIs 14–17, 49–52); primary visual and medial visual areas (V1&Vm, ROIs 18–21, 43–46); lateral visual and auditory areas (Vl&A1, ROIs 22–25, 47–50); **Fig 6E**) and used them separately for GRU training. The decoder performances were 0.869 ± 0.037 in M2&M1, 0.966 ± 0.030 in FL&HL, 0.776 ± 0.097 in PT&RS, 0.793 ± 0.060 in V1&Vm, and 0.798 ± 0.058 in Vl&A1 (mean ± SD, n = 20 models, respectively; **Fig 6F**). Consistent with the results in Fig 5B, the decoder trained with FL&HL classified behavioral states with the highest accuracy. The superior performance of FL&HL areas was also observed for decoding the locomotion speed (**S5 Fig**). Moreover, the motor area’s decoder outperformed other cortical areas except for FL&HL. The correlation of the cortical activities with dynamics of behavioral states was weakly positive in all areas (mean ± SD; 0.21 ± 0.10, n = 50 ROIs; **S6 Fig**), which could not explain the predominance of the somatosensory limb areas in the GRU decoders.

In summary, our methods accurately classified mouse behavioral states from cortex-wide functional images consistent across mice and identified the essential features of cortical areas for behavioral classification in deep learning with both CNN and RNN. These results suggest the possibility of generalized neural decoding of voluntary behaviors with a whole-body motion from the cortical activity and the generation of interpretable decoders by end-to-end approach.

## Discussion

### Advantages of end-to-end behavior decoding from cortical calcium imaging

The present study demonstrated that deep learning using CNN-based end-to-end approaches accurately decoded the mouse behavioral states from cortical activity measured by mesoscopic calcium imaging. Recently, attempted speech and handwriting movements have been decoded on the temporal scales in real-time from the cortical activity obtained by microelectrode array and electrocorticography (ECoG) from human patients [5,29,30]. Compared with the electrical recordings, calcium imaging is temporally slow but spatially high with a variable range of resolution from synaptic and cellular to regional scales. In CNN-RNN decoders, the robust performance of behavior classification was obtained using an input window from 0.067 s to 0.5 s. Our results indicate that the high spatial resolution of the calcium imaging contains sufficient information for decoding the mouse behavior even in the sub-second temporal order.

Furthermore, we visualized the most critical brain areas, the somatosensory cortex limb areas, for behavioral classification by the CNN-based end-to-end approach. These areas were commonly detected in the CNN-RNN decoders, suggesting that models were generalized between mice. Regional cortical activity in the somatosensory areas contributed to the decoding performance, supported by the RNN decoders. The somatosensory cortices were also listed in one of the essential areas in our previous study [14]. However, in the present study, mouse behavioral states were accurately classified using information only in this area, suggesting that the somatosensory cortex is the area that contributes the most to behavioral classification from cortical activity. Since mice receive sensory inputs from the left and right limbs when moving on and touching the treadmill, the regional activity in the somatosensory areas may be reflected as a featured cortical response during locomotion. In addition, the primary somatosensory cortex also receives prior information about future movements from the primary motor cortex [31]. Utilizing the neural information from input-output relationships, such as the motor and somatosensory cortices, improves the performance of robotic arm control [32]. Our interpretable approach for deep learning decoders may help to identify multiregional cortical activities related to behavioral expressions.

### Combination of CNN and RNN for behavior decoding

Recently, a convolutional and recurrent neural network model has been applied to decoding finger trajectory from ECoG data, in which CNN was used to extract the features, and LSTM was used to capture the temporal dynamics of the signal [16]. Similar to this architecture, our decoder with CNN-RNN effectively worked for mouse behavior classification and was superior to the decoder with CNN alone. Furthermore, the architecture LSTM followed by CNN was also applied to decoding the brain activity using EEG by reconstructing the visual stimuli, and it performed more accurately than the architecture CNN followed by LSTM [33]. The direction of architectures should be considered as a critical factor in the case of the combination of deep learning methods. By expanding the application of these methods in neuroscience research, behavior decoding from brain activity can deal with more complex patterns of behaviors with high temporal information, leading to the further development of BCI technologies.

## Materials and methods

### Datasets

We used the previously reported dataset, including the 18,000-frame images of fluorescent signals in the cortex measured by mesoscopic 1-photon calcium imaging at 30 frames/second and the time-matched behavioral states of locomotion and rest from head-fixed mice [14]. The dataset contains 64 sessions (for 10 min/session) from five Emx1G6 mice. The number of sessions in each mouse was 11, 12, 14, 15, and 12. We used all images (128 × 128 pixels × 18,000 frames × 64 sessions) for deep learning decoding with CNN and RNN. For deep learning analysis, we divided the five mice into subgroups at the rate of 3:1:1 for training, validation, and testing, respectively, to perform cross-validation, generating the twenty models in total (four models for each testing).

For behavioral labeling, the frames with a locomotion speed more significant or less than 0.5 cm/s were defined as a state of “Run” or “Rest,” respectively.

### Data analysis

#### Deep learning with CNN-RNN

Deep learning with CNN-RNN was performed using Python 3.6, Anaconda Packages, PyTorch (https://pytorch.org), and fastai (https://docs.fast.ai). We used a PC equipped with Ubuntu 18.04 OS and NVIDIA GeForce RTX3090 GPU. All images were normalized by subtracting the average intensity in each pixel. The normalized images were divided by the variance of intensities of all pixels. For CNN classification, all images were then converted to an RGB image *I*_*t*_ by combining three consecutive images from one frame before (red, *t* −1) to one frame after (blue, *t* +1) the target image *t* (green) with labeling a behavioral state of the target image *t* (Fig 2A). As the architecture of CNN, EfficientNet B0 was used from the Python package in GitHub (https://github.com/lukemelas/EfficientNet-PyTorch) [22].

First, we trained the CNN to classify the behavioral state from the RGB images along the data allocation (Fig 1D). For the initial values of the CNN, we used the publicly available model that was pre-trained by ImageNet [34]. We used the random batches of size 512 using Adam optimizer (https://keras.io/api/optimizers/adam/ [25]), binary cross-entropy loss function, and one-cycle training with a maximum learning rate of 0.001. In the CNN architecture, 1,280 features were extracted and fully connected to an output node. The activation function of the output node was set as sigmoid for binary classification of behavior labels. The number of epochs was set to 3 because CNN learning was efficiently progressing even in one epoch (Fig 2) and due to the capacity of the computer’s GPU. The model with the lowest loss in the validation data was adopted.

Next, a two-step training with CNN and RNN was performed for behavior state classification. Following the CNN training (Step 1), in which the initial values were set to the CNN models trained at the first stage, the RNN was trained using input data of sequential RGB images (Step 2). The inputs of RGB images for CNN were initially eleven consecutive images ranging from 0.17 s before (*I*_*t*_ −5) to 0.17 s after (*I*_*t*_ +5) the image *t*, which was labeled with the behavioral state at image *I*_*t*_ (Fig 2A). After the convolution layer of CNN, 1,280 features per image were extracted by compression with average pooling and recursive input to RNN. The GRU and LSTM were used as the RNN architectures, which consisted of 128 units, 2 layers, and a dropout of 0.2. The hyperbolic tangent function was used as an activation function for RNN. The RNN units in the second layer were then fully connected to an output node. The activation function of the output node was set to sigmoid for the binary classification of behavior labels. We used the random batches of size 32 using Adam optimizer, binary cross-entropy loss function, and one-cycle training with the maximum learning rate of 0.001. The number of epochs was set to 3. The mixed precision (https://docs.fast.ai/callback.fp16.html) was used to improve the efficiency of the two-step training. We evaluated the loss for each Epoch and adopted the model with the lowest loss in the validation data. To compare the size of the input data for the CNN-RNN classification, we tested four different lengths of the time window, i.e., 0.067 s (*t* ±2), 0.17 s (*t* ±5), 0.33 s (*t* ±10), and 0.5 s (*t* ±15) before and after the image *t* (Fig 2F). The decoder performance was evaluated by the area under the receiver operating characteristic curve (AUC) for the classification of the test data. The mean ± SD values of the decoder performance are shown in S1 Table.

#### Cut-out importance

We quantified the critical areas of images that contributed to the behavioral classification in the CNN-RNN decoder. The image (128 × 128 pixels) was divided into a 32-pixel square with a 16-pixel overlap, and each end was connected to the opposite end, thus obtaining 64 compartments. Before evaluating CNN-RNN decoders, all pixels in a compartment were masked with a value of 0. We then tested the CNN-RNN by excluding information in the masked compartment area. Each compartment was scored by importance score, calculated by subtracting the AUC using the decoder tested with the masked data from the AUC using the decoder with the unmasked data.


$$Importance\;score={AUC}_{base}-{AUC}_{masked}$$

The importance score indicates how much the decoder performance using masked data (AUC_masked_) decreased compared to unmasked data (AUC_base_). The importance scores at one-fourth of the 32-pixel square were averaged among four times overlaps at the different masked areas and plotted on an 8×8 heat map. Then, the heat maps were averaged across all models. We named this analysis “cut-out importance.”

We calculated cut-out importance and AUC by occluding the middle left and right hemispheres corresponding to the somatosensory forelimb and hindlimb areas (left, X: 49–80, Y: 17–32 pixels; right, X: 49–80, Y: 97–112 pixels; same as a 32-pixel square size) to examine whether these areas significantly affect decoding performance (S3 Fig).

#### Preprocessing of regional cortical activity

This analysis was performed using MATLAB (MathWorks). The changes in cortical activity were calculated from fluorescent signals at the 50 regions of interest (ROIs) in the cortex (25 ROIs in each hemisphere, S2 Fig), which was represented by dF/F, a percentage of changes from the baseline fluorescence (Nakai et al., 2023). In this study, a 1,000-frame moving average of dF/F was subtracted from dF/F to attenuate baseline variation of the fluorescent changes, which was an optimal filter size (S3 Fig).

#### Deep learning with RNN

Deep learning with recurrent neural network (RNN) was performed using Python 3.6 (https://www.python.org/), Anaconda Packages (https://docs.anaconda.com/anaconda/packages/old-pkg-lists/2021.05/py3.6_win-64/), TensorFlow (https://www.tensorflow.org/) and Keras (https://keras.io/). A PC with Ubuntu 16.04 OS and NVIDIA GeForce RTX2080 GPU was used. The code for deep learning is available in the following GitHub repository (https://github.com/atakehiro/Neural_Decoding_from_Calcium_Imaging_Data).

For binary classification of behavioral states, we assigned a value of 1 and 0 to the frames labeled “Run” and “Rest,” respectively. The input data for RNN models was 31 frames of the preprocessed dF/F, which localized from 15 frames before to 15 frames after a behavior-labeled frame, and a one-frame sliding window was used to cover all except for the first and last 15 frames. This period ranged up to 0.5 s after the behavioral expression had been used in the previous study [5]. Each input data was normalized by Min-Max Scaling. We used six RNN architectures (simple RNN, LSTM, GRU, and their bidirectional counterparts) for behavior classification in the same manner. The model was trained with the random batches of size 256 using Adam optimizer [25] and binary cross-entropy loss function. The unit number of RNN was set to 32. The hyperbolic tangent function was used as an activation function. The RNN is followed by a one-node fully connected layer. The activation function of the last classification node was set to sigmoid for the binary classification of behavior labels, and the label smoothing was set to 0.01. The number of epochs was set to 30, in which the models reached a stable loss and accuracy for the training and validation data. The model in the epoch with the lowest loss in the validation data was adopted. As a control, we generated the models trained with the behavioral labels permuted randomly (Random) and the models trained with non-preprocessed dF/F (Raw). The decoder performance was evaluated by the AUC for the classification of the test data. The mean ± SD values of the decoder performance are shown in S1 Table.

#### Analysis of temporal differences in the input window using RNN decoders

To investigate the optimal conditions, we compared GRU decoders trained using the different lengths of the input time window and the temporally shifted target labels of behavioral classification (Fig 5). The target labels have temporally shifted the position from the center of the time window in the ranges from −2 to 2 s (from −60 to 60 frames) at 10-frames steps. The lengths of time window size 5, 10, 15, 20, 25, and 30, and the shifts of target label -60, -50, -40, -30, -20, -10, 0, 10, 20, 30, 40, 50, and 60 were analyzed.

#### Deep SHAP

We used Deep SHAP (the SHAP Python package in GitHub (https://github.com/slundberg/shap)) to visualize the basis for deep learning classifications. Deep SHAP is one of the feature attribution methods designed by combining SHAP (SHapley Additive exPlanation), which assigns each feature an importance value for machine learning predictions, with DeepLIFT, which is an additive feature attribution method that satisfies local accuracy and missingness [20]. In this analysis, we randomly selected 10,000 frames from the test data (total 198,000–270,000 frames/test) to calculate SHAP values of each ROI, indicating the extent of contribution to the model output. The absolute SHAP values were averaged and represented as the overall importance of each ROI.

### Speed prediction

The deep learning architecture comprises a two-RNN layer and a fully-connected 3-layer. Two GRUs with 64 units in the first layer and 32 units in the second layer were used as the RNN layer. After RNN, data was sent to three fully connected layers, each with 16, 8, and 1 unit (s). Dropout was placed before the fully connected layers, and the ratio was set to 0.3, 0.2, and 0.1 from the side closer to the RNN layer. In addition, batch normalization was placed before dropout. Rectified linear unit (ReLU) was used as the activation function for the first two fully connected layers on the RNN side, and linear was used as the activation function for the final layer. The loss function was set to mean squared error (MSE), and other training settings were the same for behavior classification. The coefficient of determination, R^2^, was calculated by regression score function (sklearn.metrics.r2_score). The mean absolute error (MAE) was used to assess the decoding performance.

### Linear regression model

The Python scikit-learns library was used for the linear model. Preprocessed dF/F of 50 cortical regions were used as input to estimate the behavioral states and speed at the time point. Logistic regression (sklearn.linear_model.LogisticRegression) was used for behavior classification, and linear regression (sklearn.linear_model.LinearRegression) was used to estimate speed. Models were trained on training data, and decoding performance was evaluated on test data. Validation data was not used for the linear model.

### Statistics

All statistical analyses were conducted in MATLAB (MathWorks). All bar plots with error bars represent mean ± SD. All box plots represent the median with interquartile range (IQR) (box) and 1.5 × IQR (whiskers), gray lines indicate the line plot of individual results, and ’o’ symbols indicate the outlier. For all statistical tests, the normality of the data and equal variance of groups were not assumed, and non-parametric tests were used for group comparisons. Wilcoxon rank-sum test with Holm correction was used. The significance level was set to *P* < 0.05.

## Supporting information

S1 Table List of the decoder performance. (XLSX)

S1 Fig Importance scores in each session. Importance scores in each session were overlaid on the images of calcium imaging from each mouse.
(TIF)

S2 Fig Fluorescent calcium signals and corresponding cortical areas. (A) An image of cortical fluorescent signals with the 50 ROIs whose numbers correspond to the cortical areas based on the mouse brain atlas[14]. (B) Representative traces of the behavioral states (top) and the cortical activities (bottom). Cortical activity was represented by fluorescent changes at each cortical area. Behavior states were defined by locomotion speed (0.5 cm/s). Images and locomotion speed were measured at 30 frames per second during a 10 min session.
(TIF)

S3 Fig Importance of the somatosensory areas for behavioral classification. (A) Representative images of an all-pixel image (left) and an image occluding subdivisions of the middle left and right hemispheres corresponding to the somatosensory cortex forelimb and hindlimb areas (right). (B) The area under the receiver operating characteristic curves (AUC) of the decoders using all-pixel images and occlusion images. ***P < 0.001, Wilcoxon rank-sum test, n = 20 models.
(TIF)

S4 Fig Preprocessing of the fluorescent signals for deep learning classification. (A) To suppress baseline variation, the raw fluorescent signal (dF/F) at each ROI was subtracted by its moving average, which was calculated within the frames defined by filter size. (B) Preprocessing data contributed to the improvement of the performance of GRU decoders. *P < 0.05, **P < 0.01, ***P < 0.001, Wilcoxon rank-sum test with Holm correction, n = 20 models.
(TIF)

S5 Fig Speed decoding from regional cortical activity. (A) Representative traces for speed decoding using GRU. The speed of real mouse movement (locomotor activity) detected by the treadmill was shown in a blue trace (Ground Truth). The predicted speed was shown in an orange trace (Prediction). (B) The coefficient of determination (R^2^) of the GRU and linear regression (LR) models. (C) The mean absolute error (MAE) of the GRU and LR models. ***P < 0.001, Wilcoxon rank-sum test, n = 20 models. (D) The R^2^ of the GRU models using cortical activity from M2&M1, FL&HL, PT&RS, V1&Vm, and Vl&A1. The convention of cortical areas is the same as Fig 6. ***P < 0.001, Wilcoxon rank-sum test with Holm correction, n = 20 models each. (E) The MAE of the GRU models using cortical activity from M2&M1, FL&HL, PT&RS, V1&Vm, and Vl&A1. ***P < 0.001, *P < 0.05 Wilcoxon rank-sum test, n = 20 models each.
(TIF)

S6 Fig Correlation between fluorescent signals and locomotor activity. The graph shows the values of Pearson’s correlation coefficient between the fluorescent signals in ROIs and the binarized behavior states (mean ± SD, n = 64 sessions). All ROIs have a weak or moderate positive correlation with locomotor activity.
(TIF)
