# Supplementary figures and images for: End-to-end deep learning approach to mouse behavior classification from cortex-wide calcium imaging
Source: PLoS Comput Biol. 2024 Mar 13;20(3):e1011074. doi: 10.1371/journal.pcbi.1011074 (PMC10986998; doi:10.1371/journal.pcbi.1011074)

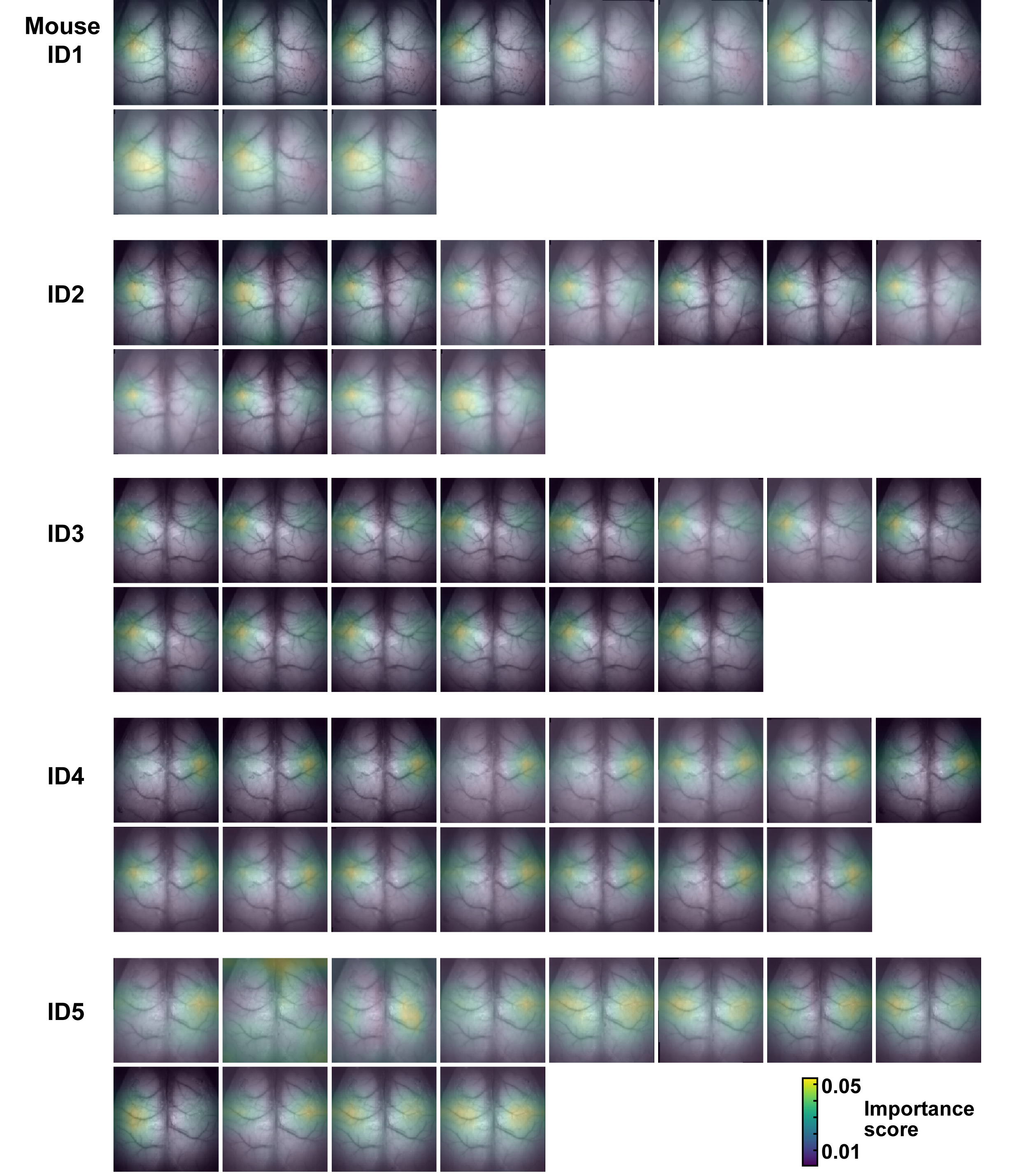

Supplement: S1 Fig — Importance scores in each session were overlaid on the images of calcium imaging from each mouse. (TIF) [file pcbi.1011074.s002.tif]

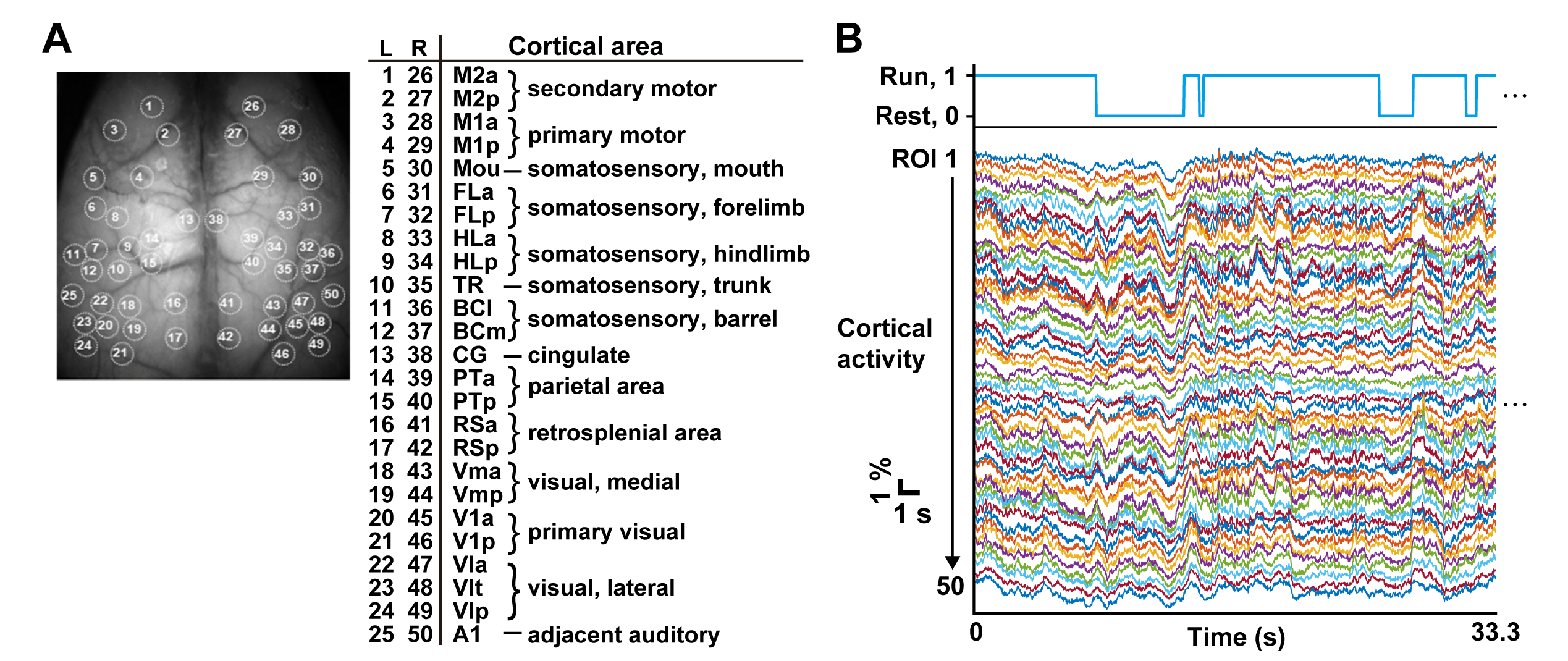

Supplement: S2 Fig — (A) An image of cortical fluorescent signals with the 50 ROIs whose numbers correspond to the cortical areas based on the mouse brain atlas[14]. (B) Representative traces of the behavioral states (top) and the cortical activities (bottom). Cortical activity was represented by fluorescent changes at each cortical area. Behavior states were defined by locomotion speed (0.5 cm/s). Images and locomotion speed were measured at 30 frames per second during a 10 min session. (TIF) [file pcbi.1011074.s003.tif]

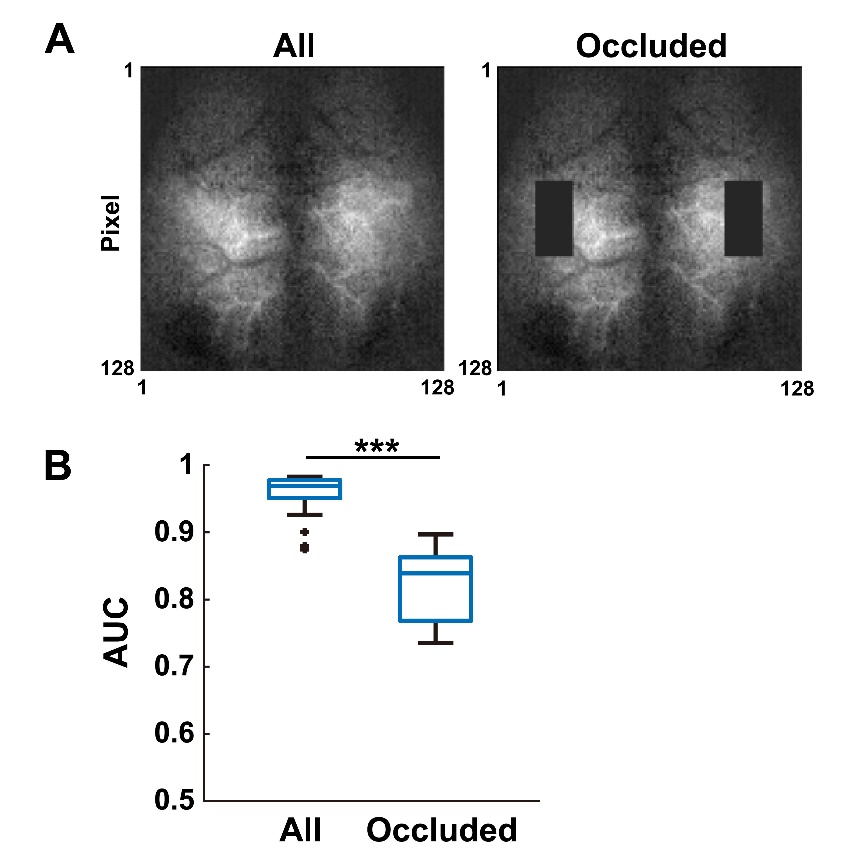

Supplement: S3 Fig — (A) Representative images of an all-pixel image (left) and an image occluding subdivisions of the middle left and right hemispheres corresponding to the somatosensory cortex forelimb and hindlimb areas (right). (B) The area under the receiver operating characteristic curves (AUC) of the decoders using all-pixel images and occlusion images. ***P < 0.001, Wilcoxon rank-sum test, n = 20 models. (TIF) [file pcbi.1011074.s004.tif]

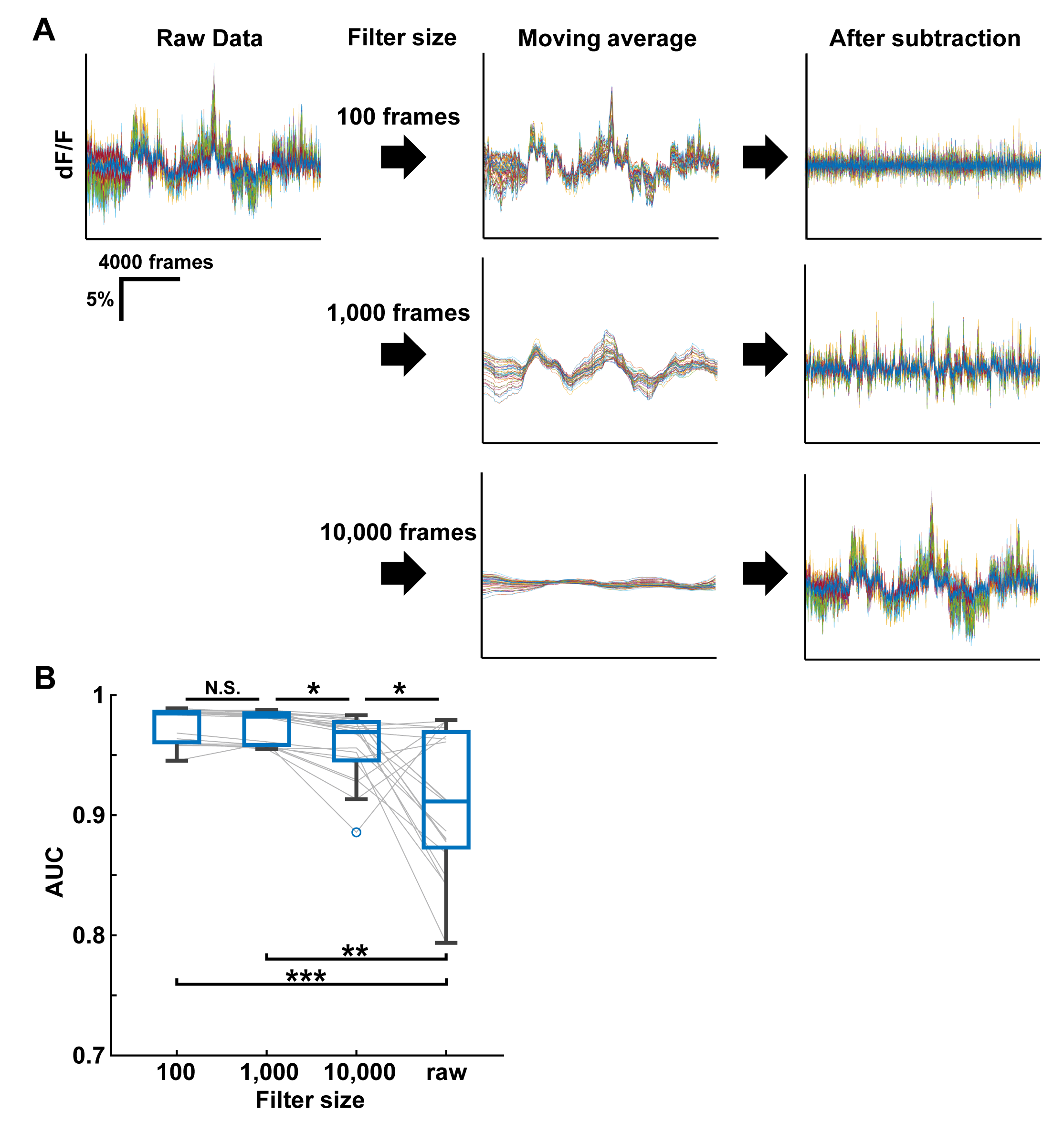

Supplement: S4 Fig — (A) To suppress baseline variation, the raw fluorescent signal (dF/F) at each ROI was subtracted by its moving average, which was calculated within the frames defined by filter size. (B) Preprocessing data contributed to the improvement of the performance of GRU decoders. *P < 0.05, **P < 0.01, ***P < 0.001, Wilcoxon rank-sum test with Holm correction, n = 20 models. (TIF) [file pcbi.1011074.s005.tif]

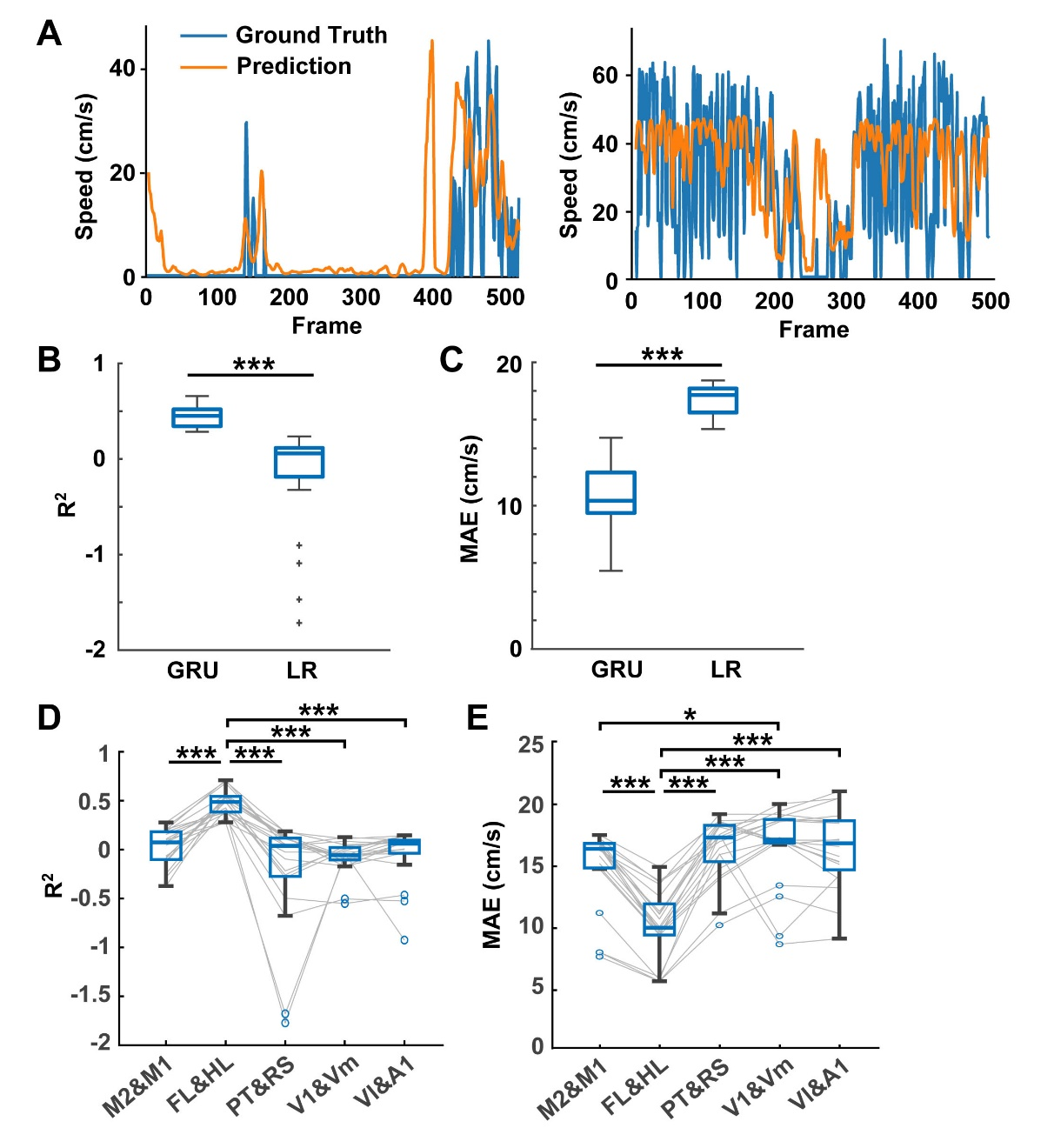

Supplement: S5 Fig — (A) Representative traces for speed decoding using GRU. The speed of real mouse movement (locomotor activity) detected by the treadmill was shown in a blue trace (Ground Truth). The predicted speed was shown in an orange trace (Prediction). (B) The coefficient of determination (R2) of the GRU and linear regression (LR) models. (C) The mean absolute error (MAE) of the GRU and LR models. ***P < 0.001, Wilcoxon rank-sum test, n = 20 models. (D) The R2 of the GRU models using cortical activity from M2&M1, FL&HL, PT&RS, V1&Vm, and Vl&A1. The convention of cortical areas is the same as Fig 6. ***P < 0.001, Wilcoxon rank-sum test with Holm correction, n = 20 models each. (E) The MAE of the GRU models using cortical activity from M2&M1, FL&HL, PT&RS, V1&Vm, and Vl&A1. ***P < 0.001, *P < 0.05 Wilcoxon rank-sum test, n = 20 models each. (TIF) [file pcbi.1011074.s006.tif]

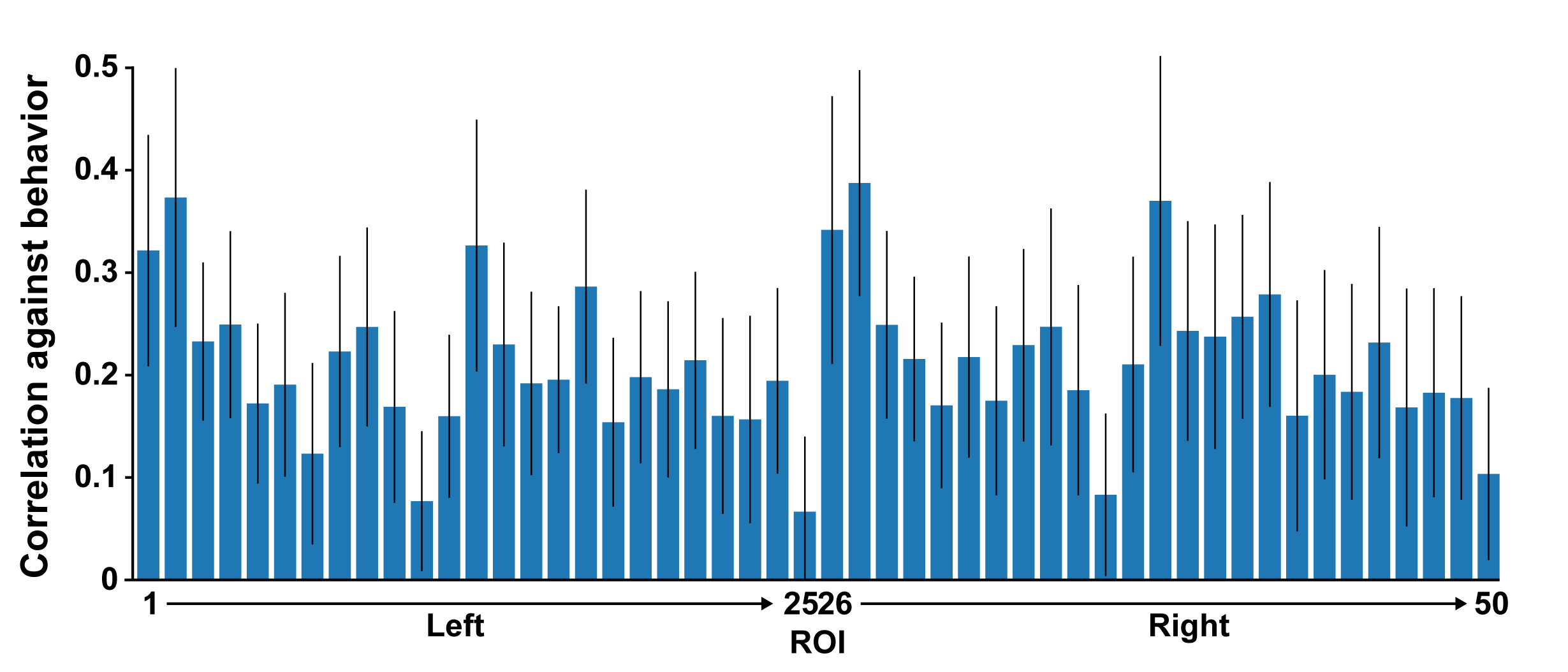

Supplement: S6 Fig — The graph shows the values of Pearson’s correlation coefficient between the fluorescent signals in ROIs and the binarized behavior states (mean ± SD, n = 64 sessions). All ROIs have a weak or moderate positive correlation with locomotor activity. (TIF) [file pcbi.1011074.s007.tif]
